# Basal Endogenous Steroid Hormones, Sex Hormone-Binding Globulin, Physical Fitness, and Health Risk Factors in Young Adult Men

**DOI:** 10.3389/fphys.2018.01005

**Published:** 2018-07-27

**Authors:** Sheila S. Gagnon, Bradley C. Nindl, Jani P. Vaara, Matti Santtila, Keijo Häkkinen, Heikki Kyröläinen

**Affiliations:** ^1^Neuromuscular Research Center, Biology of Physical Activity, Faculty of Sport and Health Sciences, University of Jyväskylä, Jyväskylä, Finland; ^2^Wolf Orthopaedic Biomechanics Laboratory, Department of Health and Rehabilitation Sciences, The University of Western Ontario, London, ON, Canada; ^3^Neuromuscular Research Laboratory/Warrior Human Performance Research Center, Department of Sports Medicine and Nutrition, University of Pittsburgh, Pittsburgh, PA, United States; ^4^Department of Leadership and Military Pedagogy, National Defence University, Helsinki, Finland; ^5^Personnel Division, Defence Command, Finnish Defence Forces, Helsinki, Finland

**Keywords:** testosterone, sex hormone-binding globulin, cortisol, waist circumference, cardiovascular health, aerobic capacity, physical fitness

## Abstract

**Purpose:** Few large-scale population-based studies have adequately examined the relationships between steroid hormones, health status and physical fitness. The purpose of the study was to describe the relationship of serum basal endogenous steroid hormones (testosterone, TES; empirical free testosterone, EFT; cortisol, COR) and sex hormone-binding globulin (SHBG) to body composition, cardiovascular risk factors, and physical fitness in young healthy men.

**Methods:** Male reservists (25 ± 4 years, *N* = 846) participated in the study. Basal TES, EFT, COR, and SHBG were measured in morning fasted blood. Stepwise regression analyses were used to examine associations between individual hormones to four separate categories: (1) body composition; (2) cardiovascular risk factors; (3) relative, and (4) absolute physical fitness.

**Results:** Higher TES, EFT, and SHBG were associated with lower waist circumference (TES: β = -0.239, *p* < 0.001; EFT: β = -0.385, *p* < 0.001), % body fat (TES: β = -0.163, *p* = 0.003), and body mass index (SHBG: β = -0.435, *p* < 0.001). Lower cardiovascular risk factors were associated with higher TES, EFT and SHBG concentrations, especially between SHBG and triglycerides (β = -0.277, *p* < 0.001) and HDL (β = 0.154, *p* < 0.001). Greater maximal relative aerobic capacity was concurrent with higher TES, EFT, and SHBG (β = 0.171, 0.113, 0.263, *p* < 0.001, =0.005, <0.001, respectively).

**Conclusion:** Higher basal concentrations of TES, EFT, and SHBG were weakly associated with healthier body composition, fewer cardiovascular risk factors and greater relative aerobic capacity in healthy young men. It would be interesting to investigate whether these relationships are still evident after a few decades, and how different training modes (endurance, strength or their combination) positively affect physical fitness, body composition and their regulatory mechanisms over the decades.

## Introduction

Testosterone has been associated with various health, lifestyle, and physical performance parameters in a variety of populations, inclusive of clinical and active/healthy populations. For example, Bhasin et al. (2001) reported favorable relationships of exogenous TES administration on body composition, physical performance and plasma lipids in healthy young men. Additionally, a novel study by Bosco et al. (1996) reported a positive association between basal TES concentration and performance capacity (higher maximal vertical jump, faster 30 m dash and better aerobic performance) in soccer players. While exercise-induced responses of endogenous hormonal concentrations have received considerable attention in the literature, a paucity of cross-sectional studies exists for basal endogenous TES and health and fitness outcomes for young, healthy populations.

Testosterone is known to have significant anabolic effects via nitrogen retention (Bhasin et al., 2003), neuromuscular transmission (Storer et al., 2003), and potentiating the effectiveness of other hormones such as growth hormone (Giustina and Veldhuis, 1998), which can lead to favorable body composition profiles (increased FFM and decreased FM). Nonetheless, a clear relationship between body composition, strength and TES has yet to be validated with endogenous TES in a normal, heterogeneous, healthy male population. Meanwhile, improved aerobic performance capacity can be observed in men with lower basal TES as demonstrated by Bosco et al. (1996) who showed that in similarly trained athletes, those with lower basal TES performed better on the Cooper’s 12-min run. Similarly, reduced basal TES, even as low as subclinical levels, is exhibited in highly aerobically trained individuals (Daly et al., 2005). In addition to relationships to physical performance and body composition, TES has also been suggested to be associated with various health outcomes; including blood glucose and lipids, as well as BP, although inconsistent and conflicting observations have been reported (Khaw and Barrett-Connor, 1991; Muller et al., 2003; Svartberg et al., 2004; Agledahl et al., 2008). Furthermore, while not always the case, hypogonadism and TES deficiency have been associated with CVD, insulin resistance, diabetes mellitus, and the metabolic syndrome (Su et al., 2014). Thus, higher basal TES concentration would seem to contribute to more favorable blood lipid profiles and glucose concentration as well as healthier BP levels.

While the scope of this study examines endogenous steroid hormones, it is nonetheless important to mention the existence of exogenous steroid hormones, especially the abuse of anabolic-androgenic steroids (AAS), and their profound impact on health. In some diseases, AAS are administered therapeutically, however they are also frequently used in excess to enhance physical performance, and aesthetic purpose (Albano et al., 2017; Bertozzi et al., 2017; Salerno et al., 2018). This is not only seen in athletes, but in prepubescent (Shokri et al., 2009), adolescent (Shokri et al., 2009; Albano et al., 2017; Bertozzi et al., 2017) and the general population in fitness centers as well, and has been even considered a “serious public health concern” (Salerno et al., 2018) and a world-wide pandemic (Pope et al., 2014; Bertozzi et al., 2017). Whole-body organ damage has been documented from AAS abuse (Albano et al., 2017) and is linked to behavioral disorders (Bertozzi et al., 2017) and cancer, particularly of the Leydig cells (Salerno et al., 2018). One of the most frequently abused AASs in the world is Nandrolone, a synthetic derivative of testosterone, actually decreases the production of endogenous testosterone by reducing the conversion of cholesterol to pregnenolone (Svechnikov et al., 2010; Salerno et al., 2018).

With an interplay of factors influencing biological activity of TES, two of these important factors are SHBG, which specifically binds circulating TES, and COR the antagonist to TES. Although SHBG has a crucial influence on circulating bioavailable TES, few studies have considered it as a primary contributor to health status (Gyllenborg et al., 2001; Bataille et al., 2005; Agledahl et al., 2008). Nevertheless, stronger associations have been observed between blood lipids and SHBG than with TES (Bataille et al., 2005). SHBG levels are modulated by sex hormones (Anderson, 1974; Bataille et al., 2005) and a marked increase is observed in men after the age of 50 (Vermeulen et al., 1972; Anderson, 1974). An increase in SHBG following a physical training regimen has been observed in military training studies lasting 8 – 12 weeks (Tanskanen et al., 2011; Vaara et al., 2015; Drain et al., 2017). Hammami et al. (2017) also showed higher SHBG levels in elite soccer players over two seasons when compared to controls. Meanwhile, the catabolic action of COR contributes in an antagonistic fashion to that of TES for muscle tissue remodeling. An inverse relationship has been suggested for COR and aerobic capacity in similarly trained individuals (Bosco et al., 1996). COR is often used as a marker for over-reaching/overtraining and excessive stress in athletes (Kraemer et al., 2004; Hammami et al., 2017) and in military training (Tanskanen et al., 2011) and a reduction in performance is observed. Evidence of a relationship between elevated COR concentrations and elevated total cholesterol (CHOL) in individuals with CVD, in particular with greater severity of CVD, as well as with moderate CAD, and SBP greater than 160 mmHg (Troxler et al., 1977; Filipovsky et al., 1996; Phillips et al., 1998), has previously been demonstrated. Whether this relationship is already evident in a young healthy population can provide insight on future health risks.

The importance of studying basal concentrations of endogenous TES, COR, and SHBG resides in their significance in mediating health and fitness outcomes (e.g., Nindl et al., 2001), and could provide an indication of health risk for future health problems such as CVD and CAD (e.g., Su et al., 2014). Few large-scale population-based studies have adequately examined such relationships. Therefore, the purpose of the present study was to describe the relationship of body composition, cardiovascular (CV) risk factors, and physical fitness profile to basal endogenous steroid hormones (TES and COR) and SHBG to in a large cohort of healthy men. We hypothesized that men with higher basal concentrations of TES would have a healthier body composition, lower CV risk factors and better performance for physical fitness parameters of muscular strength, endurance and agility. In contrast, men with higher basal concentrations of COR would show less desirable body composition, greater CV risk factors and reduced performance for physical performance, with the exception of aerobic fitness.

## Materials and Methods

### Participants

The present study was a cross-sectional study consisting of young Finnish men. Inclusion criteria consisted of all reservists who were called up to military refresher training and deemed physically fit to participate by a physician (see Protocol, below). A total of 1155 reservists were called up, in a total of 8 testing sessions. 846 men between the ages of 18 and 48 years (25.1 ± 4.7 years; height 1.8 ± 0.1 m; body mass 80.5 ± 13.5 kg) volunteered to be a part of the study group (see **Table 1**). Exclusion criteria included reporting any of the following: respiratory or CVD, diseases or injuries causing restrictions for physical activity, chronic musculoskeletal disease or injury, blood pressure medication, infection during the last 2 weeks, chest pain during exercise, and being female. Prior physical activity level was surveyed by a questionnaire and has been reported in a previous paper (Vaara et al., 2014a). They categorized physical activity into leisure-time, commuting, and occupational activity and reported the percentage of participants who responded to have low, moderate and high levels for each category. 29.7, 39.8, and 30.4% of participants self-reported leisure-time physical activity as low, moderate and high, respectively. Data was analyzed for valid cases, see results tables for respective *N* included in individual analyses. Participants were not undergoing specific military training prior to the study. Participants provided written informed consent. This study was approved by the Ethical Committee of the Central Finland Health Care District, Jyväskylä, Finland, and the Surgeon General of the Finnish Defence Forces.

**Table 1:** Descriptive data of study population.

	*N*	Mean	*SD*	IQR	
				Q1	Q3
Physical characteristics					
Age (years)	846	25	5	22	26
Height (m)	844	1.80	0.06	1.76	1.84
Body mass (kg)	842	80.6	13.4	71.7	87.8
Hormones and binding protein					
TES (nmol^.^L^-1^)	827	17.4	5.0	13.7	20.3
EFT (nmol^.^L^-1^)	825	280.39	85.96	216.70	330.32
COR (nmol^.^L^-1^)	827	488	124	403	571
SHBG (nmol^.^L^-1^)	825	34.0	12.3	25.5	40.9
Body composition					
BMI (kg^.^m^-2^)	842	24.8	3.8	22.3	26.8
WC (cm)	844	86.3	10.4	79.0	91.3
FFM (kg)	839	65.4	7.4	60.3	70.0
%BF	839	17.9	7.2	12.6	22.1
Cardiovascular health					
HR (bpm)	814	66	12	58	74
SBP (mmHg)	844	123	12	115	130
DBP (mmHg)	844	77	9	71	82
Glucose (mmol^.^L^-1^)	826	5.40	0.41	5.14	5.63
CHOL (mmol^.^L^-1^)	829	4.5	0.9	3.9	5.0
Triglycerides (mmol^.^L^-1^)	829	1.03	0.53	0.68	1.20
HDL (mmol^.^L^-1^)	829	1.49	0.36	1.25	1.69
LDL (mmol^.^L^-1^)	826	2.43	0.63	1.97	2.79
CHOL/HDL	829	3.2	1.0	2.5	3.7
Relative physical fitness					
Agility run (s)	812	6.07	0.44	5.75	6.31
Sit-ups (reps^.^min^-1^)	792	38	10	31	45
Push-ups (reps^.^min^-1^)	777	29	13	20	37
Squats (reps^.^min^-1^)	773	44	9	39	50
LP_max_ (N^.^kg^-1^)	809	40.0	11	30	42
BeP_max_ (N^.^kg^-1^)	821	11	3	10	13
VO_2peak_ (ml^.^kg^-1.^min^-1^)	787	41.6	8.1	36.0	46.9
Absolute physical fitness					
LP_max_ (N)	812	2939	871	2371	3372
BeP_max_ (N)	823	900	199	759	1005
VO_2peak_ (L^.^min^-1^)	783	3.30	0.58	2.87	3.69

*Relative physical fitness is relative to the participants’ body mass in kg. Note that agility run, sit-ups, push-ups and squats are considered relative to body mass as the subject is required to move their body mass to complete the task. Means and standard deviation (SD) are presented. IQR, Interquartile Range; Q1, First quartile; Q3, Third quartile.*

### Protocol

**FIGURE 1:**
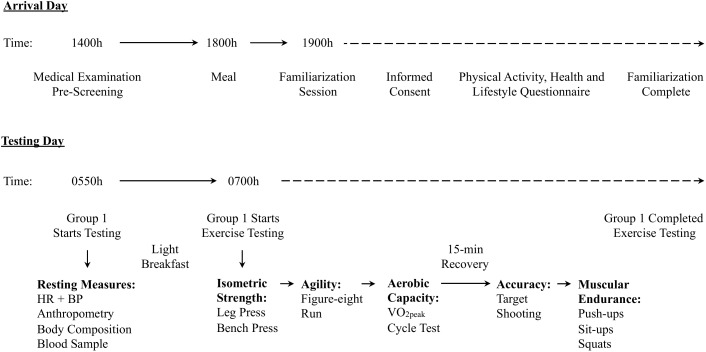
Timing and organization of the research study protocol that occurred during 8 separate testing sessions. This includes the approximate timing of events on arrival and testing day for the military training refresher course and study participation. Arrival day included medical screening, and a familiarization session. Testing day has the approximate timing of when testing started, as well as the order of exercise testing.

Participants arrived at their respective region’s garrison by 1400 h on the day prior to their specified testing date. Upon arrival, participants underwent a medical examination by a physician, as pre-screening for participation in the study. A familiarization information session detailed the purpose of the study, and explained the protocol as well as the measurements. Participants also completed a Physical Activity, Health and Lifestyle Questionnaire during the information session. Participants were divided into groups of 10, within which they would complete the testing session the following day; the first group commencing at 0550 h, subsequent groups at 10-min intervals. The timeline for a testing session is outlined in **Figure 1**. Participants underwent an overnight fast, with unrestricted water ingestion for 12 h prior to obtaining a fasting blood sample. Participants were subsequently provided a light breakfast which included a standardized serving of 1100 kcal containing 60% carbohydrates, 15% protein, 25% fat (Vaara et al., 2014b), and with a maximum of one serving of a caffeinated drink, prior to exercise testing.

### Blood Samples and Analysis

A venous blood sample was taken from the antecubital vein with the subject in a supine position. Terumo VenoSafe^TM^ (Terumo Europe, Leuven, Belgium) blood sampling method was used, and aliquoted into 5 collection tubes. K_2_EDTA whole blood collection tubes were immediately centrifuged and analyzed at the collection site. Serum samples were allowed to coagulate according to manufacturer’s instructions and were subsequently centrifuged. Plasma glucose and serum CHOL, HDL, and triglycerides were analyzed the following day.

Serum total TES, SHBG, and COR were analyzed by Immulite 1000 (Siemens Healthcare Diagnostics Products Ltd., Gwynedd, United Kingdom). The sensitivity for these assays were 0.5, 0.2, and 5.5 nmol^.^L^-1^ for TES, SHBG, and COR, respectively. Intra- and inter-assay coefficients of variance were 5.7 and 13.2% for TES, 2.4 and 6.7% for SHBG, and 4.6 and 7.6% for COR, respectively. Time of measurement for blood sampling was standardized so that the analyses represented morning fasting hormonal concentrations. Morning samples were taken between 0550 and 0800 h as TES peaking occurs approximately between 0600 and 0800 h (Gupta et al., 2000), and COR peak between 0400 and 0800 h (Veldhuis et al., 1989; Kanaley et al., 2001). Free TES was determined statistically as EFT when TES ≥ 5 nmol^.^L^-1^ using the model 2 empirical equation by Ly and Handelsman (2005), as follows:

$$\begin{array}{ccc} \text{EFT-hi} & = & –52.65+24.4\text{TT}-0.704\text{SHBG}\\ & & –0.0782\text{TT}\times \text{SHBG}–0.0584{\text{TT}}^{2} \end{array}$$

where TT represents blood total TES.

### Body Composition

Anthropometric measurements including height, body mass, and WC, were measured with a stadiometer, commercial scale, and tape measure, respectively. BMI was calculated from height and weight. Body composition analysis for %BF and FFM were estimated via bioelectrical impedance analysis (BIA) (Inbody720, Seoul, Korea). This device has previously been shown to have high test-retest reliability (ICC = 0.9995) (Gibson et al., 2008).

### Cardiovascular Risk Factors

Resting HR was measured using a monitor (Suunto Smart Belt, Finland), and later analyzed using Polar Precision Performance software (SW 4.03.043 Polar, Kempele), with the subject lying supine for 5 min. Mean resting HR was taken over the last 3 min. BP was measured twice using an automatic brachial cuff (Omron M6 Comfort, Omron Healthcare Europe B.V. Kruisweg 577–2132 NA, Hoofddorp, The Netherlands) in a seated position at 1–2 min intervals. Mean BP is reported. Blood lipids, including serum CHOL, triglycerides, HDL, and plasma glucose, were analyzed by Konelab^TM^ 20 XTi (Thermo Fisher Scientific Oy, Vantaa, Finland). The reportable range for CHOL, triglycerides, and HDL assays were 0.1 – 15, 0.09 – 11, and 0.04 – 2.84 mmol^.^L^-1^, respectively. Intra- and inter-assay coefficients of variance were 1.1 and 2.1% for CHOL, 1.0 and 3.8% for triglycerides, and 0.5 and 7.6% for HDL, respectively. LDL was calculated using the Friedewald et al. (1972) equation. Sensitivity and intra- and inter-assay coefficients of variance for glucose were 0.1 mmol^.^L^-1^, 1.0 and 2.0%, respectively.

### Physical Fitness

Standardized testing procedures were implemented for physical fitness testing in an attempt to minimize inter-rater and inter-session variability. Experienced military personnel and individuals with a major in sport and exercise testing assessed all physical fitness parameters. Testing occurred in large sports halls at military sites with ambient temperature maintained between 20 and 22°C.

Maximal isometric strength testing consisted of maximal bilateral leg press (LP_max_) and bench press (BeP_max_) exercises. Leg and bench press trials were performed on an isometric electromechanical dynamometer. LP_max_ was completed in a seated position with a knee angle of 107° (Häkkinen et al., 1985). For BeP_max_, the subject was lying supine with their back flat on a bench, and feet flat on the floor. The shoulder and elbow joints were positioned at 90°. LP_max_ and BeP_max_ trials were preceded by two submaximal trials (first 50% of individual maximum, second almost maximal effort) as a familiarization and warm-up. Participants were instructed to produce maximal force as quickly as possible, and maintain it for about 3 s. Three maximal trials were performed for both the LP_max_ and BeP_max_ exercises, with 30 s recovery between trials. Standardized motivation was given by testing personnel to each subject to encourage maximal effort. Participants were monitored closely for proper technique; trials performed incorrectly were discarded. Isometric strength outputs were collected at a sampling frequency of 1 kHz and analyzed using Signal 2.16 software.

The figure-eight agility run involved a figure-eight circuit with two pylons at a distance of 10 m apart (Tegner et al., 1986). Each subject had one familiarization trial, followed by two maximal attempts. Test time was measured using photocells (Newtest Powertimer, Newtest Oy, Oulu, Finland) that automatically recorded time to completion.

Maximal aerobic capacity was determined using a graded, multistage, maximal test to volitional fatigue on a cycle ergometer (Ergoline 800S, Ergoselect 100K, Ergoselect 200K, Bitz, Germany). The initial workload was set at 50 W, with an increase of 25 W every 2 min. The test was stopped either at volitional fatigue, or if the subject was unable to keep a pedaling cadence between 60 – 90 rpm. HR was measured throughout the maximal test using a Polar Vantage NV or S610, S710 or S810 HR monitor (Polar, Kempele, Finland) and analyzed using MILFIT 4 (Aino Active, Helsinki, Finland) software (Santtila et al., 2013).

Muscular endurance testing involved push-ups, sit-ups, and repeated squats. Participants completed as many repetitions as possible in 60 s for each exercise. There was a recovery of 5 min between tests. Standardized positioning was used for each exercise, described elsewhere (Fogelholm et al., 2006; Kyröläinen et al., 2008). Trials performed with incorrect technique were not counted.

### Statistical Analysis

Separate stepwise regression analyses were used to predict each hormone (TES, EFT, COR) and SHBG. Predictor variables were clustered in the following four categories: (1) body composition (BMI, WC, FFM, and %BF); (2) CV risk factors (HR, SBP, DBP, glucose, CHOL, triglycerides, HDL, LDL, CHOL/HDL); (3) relative (rel) physical fitness (agility run, sit-ups, push-ups, repeated squats, relLP_max_, relBeP_max_, relVO_2peak_), where relLP_max_, relBeP_max_, and relVO_2peak_ are relative to body mass; and (4) absolute physical fitness (LP_max_, BeP_max_, VO_2peak_). Multiple comparison bias was addressed using a family-wise error correction strategy. Given that four stepwise regression calculations were conducted within each family of independent variables, the threshold for including an independent variable within the prediction equation was adjusted to be *p* = 0.05/4 = 0.0125, and the threshold for statistical significance of each model was similarly adjusted to be *alpha* = 0.0125. Normality was tested using Kolmogorov–Smirnov test and histograms. When appropriate, data was transformed into natural logarithm (ln) to improve normality. This was done for TES, EFT, COR, SHBG, triglycerides, relLP_max_, relBeP_max_, LP_max_ and BeP_max_. Descriptive statistics (mean ± SD, interquartile range: Q1, Q3) for all variables were calculated. Statistical analysis was performed using SPSS (IBM SPSS for Windows Version 19.0.0).

## Results

Descriptive data of the physical characteristics of the participants, as well as hormonal and SHBG concentrations, and the variables included in the four independent grouping categories: (1) body composition; (2) CV risk factors; (3) relative physical fitness; and (4) absolute physical fitness, are shown in **Table 1**.

All hormones and SHBG (*F*_2,818_ = 71.839, *p* < 0.001; *F*_2,816_ = 50.016, *p* < 0.001; *F*_1,820_ = 191.408, *p* < 0.001; *F*_1,824_ = 23.304, *p* < 0.001, for TES, EFT, SHBG and COR, respectively) were related to body composition variables. **Table 2** displays the hierarchical regression models for body composition variables when related to TES, EFT, SHBG and COR. WC and %BF were inversely associated with concentrations of TES, and WC inversely with EFT concentrations. FFM was positively associated with EFT concentration. Endogenous TES explained 14.7% of the variation in WC and %BF, while EFT concentration explained 10.7% of the variation in WC and FFM. SHBG concentration was inversely associated with BMI, which explained 18.9% of the variation. Finally, COR and WC were inversely associated, although only explaining 2.6% of the variation.

**Table 2:** Hierarchical regression models for the prediction of endogenous steroid hormones and SHBG, with body composition variables.

	lnTES			lnEFT			lnSHBG			lnCOR		
Body Composition	R^2^_adj_ = 14.7%			R^2^_adj_ = 10.7%			R^2^_adj_ = 18.8%			R^2^_adj_ = 2.6%		
Variable	N = 821			N = 819			N = 822			N = 826		

	**Beta**		***p***	**Beta**		***p***	**Beta**		***p***	**Beta**		***p***

BMI		NS			NS		-0.435		<0.001^∗^		NS	
WC	-0.242		<0.001^∗^	-0.388		<0.001^∗^		NS		-0.166		<0.001^∗^
FFM		NS		0.129		0.002^∗^		NS			NS	
%BF	-0.164		0.003^∗^		NS			NS			NS	

*R^2^_*adj*_, adjusted R squared; Beta, standardized regression coefficient; ^∗^statistically significant, p_*in*_< 0.0125; NS, non-significant.*

All hormones and SHBG (*F*_3,787_ = 28.866, *p* < 0.001; *F*_4,784_ = 21.295, *p* < 0.001; *F*_3,819_ = 51.262, *p* < 0.001; *F*_3,791_ = 24.350, *p* < 0.001, for TES, EFT, SHBG and COR, respectively) were related to CV risk factors. **Table 3** displays the hierarchical regression models for CV risk factors when related to TES, EFT, SHBG and COR. Inverse associations of CHOL/HDL ratio, HR, and glucose, were observed with concentrations of TES and EFT. EFT also had a positive association with triglycerides. TES and EFT explained 9.6 and 9.3% of the variation in CV risk factors, respectively. SHBG was inversely associated with triglycerides and SBP, and positively associated with HDL, explaining 15.5% of the variation. COR was positively associated with SBP, HDL, and HR, explaining 8.1% of the variation.

**Table 3:** Hierarchical regression models for the prediction of endogenous steroid hormones and SHBG, with cardiovascular risk factors.

	lnTES			lnEFT			lnSHBG			lnCOR		
Cardiovascular	R^2^_adj_ = 9.6%			R^2^_adj_ = 9.3%			R^2^_adj_ = 15.5%			R^2^_adj_ = 8.1%		
Risk Factor	N = 791			N = 789			N = 823			N = 795		

	**Beta**		***p***	**Beta**		***p***	**Beta**		***p***	**Beta**		***p***

HR	-0.134		<0.001^∗^	-0.138		<0.001^∗^		NS		0.101		0.004^∗^
SBP		NS			NS		-0.096		0.003^∗^	0.196		<0.001^∗^
DBP		NS			NS			NS			NS	
Glucose	-0.113		0.001^∗^	-0.116		0.001^∗^		NS			NS	
CHOL		NS			NS			NS			NS	
lnTriglycerides		NS		0.177		<0.001^∗^	-0.291		<0.001^∗^		NS	
HDL		NS			NS		0.158		<0.001^∗^	0.169		<0.001^∗^
LDL		NS			NS			NS			NS	
CHOL/HDL	-0.200		<0.001^∗^	-0.262		<0.001^∗^		NS			NS	

*R^*2*^_*adj*_, adjusted R squared; Beta, standardized regression coefficient; ^∗^Statistically significant, p_*in*_< 0.0125.*

All hormones and SHBG (*F*_3,752_ = 28.209, *p* < 0.001; *F*_2,780_ = 30.838, *p* < 0.001; *F*_2,761_ = 46.825, *p* < 0.001; *F*_2,760_ = 14.277, *p* < 0.001, for TES, EFT, SHBG and COR, respectively) were related to relative physical fitness, but not with absolute fitness, except for SHBG (*F*_1,744_ = 6.459, *p* = 0.008). **Table 4** displays the hierarchical regression models for relative physical fitness variables when related to TES, EFT, SHBG, and COR. Greater concentrations of TES and EFT were associated with faster agility run time and greater relative BeP_max_, while relative VO_2peak_ also had a positive association with TES. SHBG was positively associated to relative VO_2peak_ and relative BeP_max_, explaining 10.7% of the variation. COR was also inversely associated with agility run and positively associated with relative VO_2peak_, but only explaining 3.4% of the variation. TES, EFT and COR were not indicative of absolute physical fitness, while SHBG only explained 0.7% of variation (absolute BeP_max_: Beta = –0.093, *p* = 0.011).

**Table 4:** Hierarchical regression models for the prediction of endogenous steroid hormones and SHBG, with relative physical fitness variables.

	lnTES			lnEFT			lnSHBG			lnCOR		
Relative Physical Fitness	R^2^_adj_ = 9.8%			R^2^_adj_ = 7.1%			R^2^_adj_ = 10.7%			R^2^_adj_ = 3.4%		
Variable	N = 756			N = 783			N = 764			N = 763		

	**Beta**		***p***	**Beta**		***p***	**Beta**		***p***	**Beta**		***p***

Agility run	-0.139		0.001^∗^	-0.179		<0.001^∗^		NS		-0.095		0.016
Sit-ups		NS			NS			NS		NS	
Push-ups		NS			NS			NS			NS	
Squats		NS			NS			NS			NS	
lnLP_max_		NS			NS			NS			NS	
lnBeP_max_	0.120		0.003	0.139		<0.001^∗^	0.110		0.004^∗^		NS	
VO_2peak_	0.144		<0.001^∗^		NS		0.268	<0.001^∗^	0.130	0.001^∗^

*Relative physical fitness is relative to the participants’ body mass in kg. Note that agility run, sit-ups, push-ups and squats are considered relative to body mass as the subject is required to move their body mass to complete the task; R^2^_*adj*,_ adjusted R squared; Beta, standardized regression coefficient; ^∗^Statistically significant, p_*in*_< 0.0125; NS, non-significant.*

## Discussion

The main findings of the present study demonstrate that favorable body composition, lower CV risk factors, and greater maximal aerobic capacity were associated with higher concentrations of basal endogenous TES, EFT, and SHBG in young healthy men. A noteworthy finding was that SHBG might also play a role as a health biomarker in addition to acting as a binding protein.

Significant stepwise regression models were observed for TES, EFT, SHBG, and COR with body composition variables. WC exhibited inverse associations with all of TES, EFT, and COR concentrations. Recently, more attention has been given to the crucial implication that WC has on cardiometabolic health risks. Although BMI is used in standard practice to categorize individuals at an elevated health risk, Janssen et al. (2004) presented evidence that WC is a better indicator of cardiometabolic health risk than BMI. Meanwhile, Klein et al. (2007) reported that in the identification of individuals with cardiometabolic risk, including CHD and diabetes, WC is a stronger predictor than BMI. The present data revealed that body fat distribution (i.e., WC), was lower in men with higher concentrations of endogenous TES, EFT, and COR, yet BMI was not. Interestingly, a meta-analysis by Corona et al. (2016b) reported that in individuals with hypogonadism, TES supplementation led to weight loss, including a one-point reduction in BMI and a 6 cm loss in WC. In the present study, TES also had a significant inverse relationship with %BF. Previous studies have reported similar relationships between basal total TES and free TES concentrations to FM (Araujo et al., 2008; Schaap et al., 2008), while negative associations observed between TES and adiposity are generally seen with central adiposity (Kaufman and Vermeulen, 2005). A meta-analysis of 59 studies revealed a pooled effect size significant reduction of FM, with an equal and opposite increase in lean mass, with TES supplementation (Corona et al., 2016a). Based on these findings, visceral and abdominal body fat deposition and quantity are associated with circulating basal endogenous TES, EFT, and COR concentrations. This pattern of central adiposity and concentration of circulating sex steroids has also been reported in a population of healthy non-obese women (Nindl et al., 2001). Whether basal hormones or current body composition drive this relationship is uncertain with the available data.

The known anabolic effect of TES on skeletal muscle growth suggests that a positive relationship exists between FFM and TES (Bhasin et al., 1997; Bhasin et al., 2003; Herbst and Bhasin, 2004; Araujo et al., 2008). This is particularly evident in pubertal boys, where increases in TES and lean mass occur in parallel (Vandewalle et al., 2014). Vandewalle et al. (2014) has shown a positive association between free TES and lean mass, as well as with muscle cross sectional area in both the radius and tibia, throughout various stages of puberty. Interestingly, this was not reflected in the present results for TES in young adult men. A study by Gates et al. (2013) also reported that total TES and free TES had no association to lean mass, yet were significantly inversely correlated with adiposity, as was evident in the current results. Further, the participants in our study belonged to a group of healthy eugonadal men, which is in contrast to other studies that examine specific populations (i.e., hypogonadal, elderly) or use of exogenous TES (Bhasin et al., 1997, 2003; Herbst and Bhasin, 2004; Araujo et al., 2008) to demonstrate the positive relationship between FFM and TES.

Sex hormone-binding globulin explained 18.8% of the variation in BMI, which was the largest standardized regression coefficient. This association may indicate that SHBG, although its primary role is its function as a binding protein for TES and other sex steroids in circulation, could also have a more direct function in affecting body composition. While it has been shown that with an increase in FM and insulin serum levels, SHBG concentration is decreased (Kaufman and Vermeulen, 2005), the biological mechanisms by which this occurs are yet to be determined. Although SHBG is regarded as a binding protein rending SHBG-bound-TES in circulation inactive, even SHBG-bound-TES can have downstream effects via a second messenger by activation of cyclic AMP (Rosner et al., 1999). There are two binding sites on SHBG, one for steroids, and the other that binds a membrane receptor. When SHBG first interacts with its membrane receptor (e.g., androgen receptor) and then a steroid (e.g., TES, dihydrotestosterone, estradiol) binds to its other receptor (Rosner et al., 1999), it allows the steroid to exert its effect on the cell without having to enter the cell itself. Rosner et al. (1999) demonstrated this effect in human prostate explants by initiating a downstream secretion of prostate specific antigen when SHBG was first bound to its membrane androgen receptor and then estradiol was introduced into the tissue. Perhaps when SHBG concentrations are reduced, this type of sequence of events does not occur sufficiently, thereby reducing the downstream effects and resulting in increased FM and circulating insulin, ultimately affecting body composition.

When regarding the body composition parameters of BMI, %BF and WC, inverse associations were present for TES, EFT and COR to WC, with TES to %BF, and SHBG to BMI. Importantly, these three parameters (BMI, %BF, WC) are all associated with the prevalence of obesity, the development of the metabolic syndrome (Bataille et al., 2005), and diabetes mellitus (Ford et al., 1997). Obesity has been shown to result in the development of insulin resistance and impaired glucose tolerance, as well as increased insulin production from pancreatic cells (Ford et al., 1997; Gapstur et al., 2002). These adverse health outcomes result in increased circulating insulin. Insulin has been reported to inhibit SHBG production by hepatoma cells *in vitro* (Plymate et al., 1988; Gapstur et al., 2002), suggesting that insulin may be an important regulating factor for SHBG *in vivo* (Plymate et al., 1988). Furthermore, due to an inhibition of SHBG from elevated insulin, a reduced SHBG concentration in circulation could further inhibit TES secretion via androgen feedback mechanisms on the hypothalamic-pituitary-gonadal axis (Gapstur et al., 2002). This would exacerbate the situation, as a reduction in TES and SHBG would further contribute to a less favorable body composition.

The results of the present study show an association of resting CV risk factors to basal TES, EFT, SHBG and COR concentrations. In accordance with previous studies (Gyllenborg et al., 2001; Bataille et al., 2005; Agledahl et al., 2008), an inverse relationship was observed between SHBG and triglycerides. In the study by Gyllenborg et al. (2001), SHBG was the best predictor of triglycerides (*R^2^* = 17%), also the case in our regression analysis model (β = –0.291, *p* < 0.001). Although no associations were observed between TES and triglycerides, a positive association was seen between EFT and triglycerides. Gyllenborg et al. (2001) reported that the free androgen index had a positive association with triglycerides. These results are in accordance with previous findings that free androgens are associated with an atherogenic lipid profile (Gyllenborg et al., 2001; Bataille et al., 2005). The importance of the role of SHBG with triglycerides is likely due to its regulation of bioavailable TES. While free androgens have been associated with an atherogenic lipid profile, a high concentration of SHBG works in an atheroprotective manner to reduce the amount of free androgens in circulation. As Bataille et al. (2005) described, the evaluation of total TES might be misleading by demonstrating both an atherogenic (e.g., free TES) and atheroprotective (e.g., SHBG-bound) effect. Thereby, the measurement of SHBG could be a better indicator of the lipid profile.

A few studies (Gyllenborg et al., 2001; Bataille et al., 2005; Agledahl et al., 2008) have observed a positive association between TES and SHBG to HDL. Gyllenborg et al. (2001) reported that SHBG was the main predictive variable of HDL (*R^2^* = 12%). The results of the present study demonstrated that SHBG was predictive of HDL (β = 0.158, *p* < 0.001). With these findings for triglycerides and HDL, we can reasonably postulate that SHBG should not simply be considered as a binding protein for TES, but as Bataille et al. (2005) suggested, it should be considered as a one of the contributors to the hormonal regulation of the blood lipid profile, although mechanisms by which this occurs are not yet evident. The association seen with SHBG and HDL might be as a result of its down-stream effect on hepatic lipase (Bataille et al., 2005). As free androgens stimulate hepatic lipase activity (Sorva et al., 1988; Perret et al., 2002), and increased hepatic lipase activity results in a reduction of HDL (Perret et al., 2002), the strong binding affinity of SHBG to TES would result in a lower amount of free TES, thereby dampening the stimulation of hepatic lipase and consequently attenuating HDL catabolism resulting in higher circulating HDL.

Fasting blood glucose levels have been reported to be higher in men with lower TES concentrations (Barrett-Connor et al., 1990; Haffner et al., 1994; Muller et al., 2003). Meanwhile, Haffner et al. (1994) observed an inverse partial correlation (–0.18, *p* < 0.05) between free TES and glucose, 2 h after a 75 g glucose load, but not with fasting glucose, and no relationship between TES and glucose. Similarly, inverse associations with TES and free TES with fasting glucose were evident in our results. Of particular interest, TES supplementation in hypogonadal men is beneficial for their glycometabolic profile, specifically on glycaemia and insulin sensitivity (Corona et al., 2016a). This effect however, is more pronounced in younger men and men with metabolic disturbances (Corona et al., 2016a). This shows that TES plays a role in glucose metabolism, likely via its’ effects on body composition. The permutations of associations seen with TES and EFT to body composition (inverse associations to WC and %BF) and to CV risk factors (inverse associations to glucose and CHOL/HDL) together all lead toward a less than optimal health profile. This is of particular concern as high fasting glucose, a higher CHOL/HDL ratio, and greater deposition of central adiposity are risk factors for obesity, CVD, diabetes, and the development of the metabolic syndrome.

Upon further examination of our results, we found that 4.2% of our participants had the metabolic syndrome as defined by the National Cholesterol Education Program-Adult Treatment Panel III (Ncep-Atpiii, 2001). SHBG concentrations of participants with the metabolic syndrome were lower than those without [3.00 ± 0.42 *vs.* 3.48 ± 0.36 nmol^.^L^-1^; *t_(*823*)_* = 7.464, *p* < 0.001]. In a study by Bataille et al. (2005), although they did not include data on fasting glucose concentration, 17.1% of their sample met the criteria for the metabolic syndrome. Their results also demonstrated that participants with the metabolic syndrome had statistically significant lower SHBG concentrations than those without. They primarily advocated the role of SHBG in the hormonal regulation of the lipid profile. In accordance with the theories presented by Bataille et al. (2005), the present study also found that along with a lower concentration of basal SHBG, participants with the metabolic syndrome also had significantly lower TES and EFT concentrations, while COR was non-significant between groups.

Resting HR was inversely associated with TES and EFT, while an inverse relationship was demonstrated between SBP and SHBG. Previous studies have shown the same relationship in BP to SHBG (Gyllenborg et al., 2001; Muller et al., 2003; Svartberg et al., 2004). Our study did not show an association of TES with SBP nor DBP. This was also evident in a meta-analysis where no effect on SBP or DBP was shown with TES supplementation in 59 randomized controlled trials evaluating various populations (Corona et al., 2016a). Most studies examining hormones and CV factors have predominantly focused on BP and less on HR. The mechanisms by which TES and EFT function to have an effect on resting HR needs to be further studied at the physiological level. TES has anti-hypertensive effects that may be a direct mechanism by modulating blood vessel resistance and arterial blood flow (Svartberg et al., 2004).

We observed positive associations for CV risk factors and COR to resting HR and SBP. This may indicate that higher basal COR concentration has an adverse association with HR and SBP. However, any observed association of COR remains complex due to its metabolic clearance rate, as well as its rate of production (Vierhapper et al., 2004). Therefore, even the modest relationships with COR observed in the present study should be considered with caution. Nonetheless, similar results have previously been reported, although this was in individuals with CVD, CAD or SBP > 160 mmHg (Troxler et al., 1977; Filipovsky et al., 1996; Phillips et al., 1998). The present results indicate that although the contribution of elevated COR concentrations to resting HR and SBP is more pronounced in hypertensive individuals, a relationship exists in apparently healthy individuals as well. This may prove to be an additional risk factor for development of future CV health problems, although whether COR concentrations are causative or concurrent with HR and SBP is not evident.

TES, EFT, SHBG, and COR were associated with relative, but not with absolute (except SHBG) physical fitness variables. Interestingly, TES and EFT showed associations with aerobic (VO_2peak_) and short-duration anaerobic (6-s agility run) activities, but not with isometric maximal strength or muscular endurance measures (see **Table 4**). Attenuated TES concentration has previously been evidenced in highly endurance-trained men (Hackney et al., 1988, 1997, 1998; Daly et al., 2005), and in untrained men as a result of endurance training (Wheeler et al., 1991). However, some studies have not observed this diminution (Fellman et al., 1985; Hackney et al., 1989; Kraemer et al., 1995). The absence of the expected TES response in these latter studies was attributed to either the initial physical condition of the participants or the type of training stimulus. The present study sample was not analogous to either of these groups. It seems that lower basal TES concentration does not suggest an inherent potential for aerobic capacity performance, indicating that it may be the high volume of aerobic training that causes a reduction in basal TES. Thus, TES and EFT as such, may not pre-determine the trainability status of endurance capacity, though high concentrations of TES and EFT can be beneficial for muscle mass, strength and power development during training in both men (e.g., Häkkinen et al., 1988) and, especially in women (e.g., Häkkinen et al., 2001). Moreover, it has been previously reported (Bosco et al., 1996) that faster running speed (*r* = 0.47) and greater power performance (maximal vertical jump, *r* = 0.43) were correlated with higher basal TES in similarly trained athletes. It has been documented in animal studies that TES is partly responsible for the development of fast twitch type II muscle fibers (Krotkiewski et al., 1980; Dux et al., 1982; Bosco et al., 1996; Viru and Viru, 2004). While the present study demonstrated that agility run time was significantly faster in men with higher basal TES and EFT concentration, possibly due to a greater number or recruitment of fast twitch type II muscle fibers, the specificity of any detailed mechanisms were not examined.

## Conclusion

Higher concentrations of serum basal endogenous TES, EFT, and SHBG were associated with healthier body composition, fewer CV risks and greater maximal aerobic capacity (in relation to body mass) in healthy young adult men. In the future, it would be interesting to investigate whether the present relationships are still evident after a few decades. Additionally, whether chronic training over the years might lead to changes in the present relationships, and how different training modes (endurance, strength or their combination) positively affect physical fitness, body composition and their regulatory mechanisms over the decades.

## Ethics Statement

The experiments comply with the current laws of the country in which they were performed. This study was carried out in accordance with the recommendations of the Ethical Committee of the Central Finland Health Care District, Jyväskylä, Finland, and the Surgeon General of the Finnish Defence Forces. The protocol was approved by the Ethical Committee of the Central Finland Health Care District, Jyväskylä. All subjects gave written informed consent in accordance with the Declaration of Helsinki.

## Author Contributions

SG and JV: data collection and analysis. SG: data interpretation and wrote the manuscript. JV, BN, HK, and KH: assisted in the interpretation of the results and the writing of the manuscript. MS: responsible officer for the project, organized funding, and commented on the manuscript. KH and HK: study design. HK: acted as scientific head of research project and lead data collection.

## Disclaimer

The opinions or assertions contained herein are the private views of the author(s) and are not to be construed as official or reflecting the views of the United States Army or the Department of Defense.

## Conflict of Interest Statement

The authors declare that the research was conducted in the absence of any commercial or financial relationships that could be construed as a potential conflict of interest.

## ABBREVIATIONS

%BF: body fat percentage
BeP_max_: maximal bilateral bench press
BIA: bioelectrical impedance analysis
BMI: body mass index
BP: blood pressure
CAD: coronary artery disease
CHOL: total cholesterol
CHOL/HDL: total cholesterol to high density lipoprotein ratio
COR: cortisol
CV: cardiovascular
CVD: cardiovascular disease
DBP: diastolic blood pressure
EFT: empirical free testosterone
FFM: fat free mass
FM: fat mass
HDL: high density lipoprotein
HR: heart rate
LDL: low density lipoprotein
LP_max_: maximal bilateral leg press
relLP_max_: relative maximal bilateral leg press
relBeP_max_: relative maximal bilateral bench press
relVO_2peak_: relative estimated peak oxygen uptake
SBP: systolic blood pressure
SHBG: sex hormone-binding globulin
TES: testosterone
VO_2peak_: estimated peak oxygen uptake
WC: waist circumference
